# EMT and Acquisition of Stem Cell-Like Properties Are Involved in Spontaneous Formation of Tumorigenic Hybrids between Lung Cancer and Bone Marrow-Derived Mesenchymal Stem Cells

**DOI:** 10.1371/journal.pone.0087893

**Published:** 2014-02-06

**Authors:** Mei-Hua Xu, Xuan Gao, Dan Luo, Xiang-Dong Zhou, Wei Xiong, Guo-Xiang Liu

**Affiliations:** Department of Respiratory Medicine, Southwest Hospital, Third Military Medical University, Chongqing, China; Cincinnati Children’s Hospital Medical Center, United States of America

## Abstract

The most deadly phase in cancer progression is metastatic conversion. Epithelial-to-mesenchymal transition (EMT) is a key process by which cancer cells acquire invasive and metastatic phenotypes. In order to spawn macroscopic metastases, disseminated cancer cells would seem to require self-renewal capability. However, the underlying mechanism defining these processes is poorly understood. One possible mechanism underlying metastasis is fusion between myeloid cells and cancer cells. In this study, we found that spontaneously-formed tumorigenic hybrids between bone marrow-derived mesenchymal stem cells (MSCs) and three different non-small cell lung cancer (NSCLC) cell lines contributed to highly malignant subpopulations with both EMT and stem cell-like properties. Hybrids lost their epithelial morphology and assumed a fibroblast-like appearance. Up-regulation of vimentin, α-smooth muscle actin (α-SMA), and fibronectin, and down-regulation of E-cadherin and pancytokeratin were observed in tumorigenic hybrids. These cells also exhibited increased expression of the stem cell marker prominin-1 (CD133) and over-expression of transcription factors OCT4, Nanog, BMI1, Notch1, ALDH1 as well as Sox2, all genes responsible for regulating and maintaining the stem cell phenotype. In addition, in spontaneously-formed tumorigenic hybrids, increased pneumosphere-forming capacity and tumor-forming ability in NOD/SCID mice were detectable. Thus, cell fusion between lung cancer cells and MSCs provides a nonmutational mechanism that could contribute to aberrant gene expression patterns and give rise to highly malignant subpopulations both capable of EMT and with properties of cancer stem cells (CSCs).

## Introduction

Lung cancer, especially non-small-cell lung cancer (NSCLC), remains the leading cause of cancer-related mortality worldwide. The most common forms of so-called NSCLC include adenocarcinoma, squamous cell carcinoma and large cell carcinoma [1], [2]. Tumor metastasis is the primary cause of death due to NSCLC. However, the mechanisms involved in tumor metastasis remain poorly understood.

The epithelial-mesenchymal transition (EMT) is a trans-differentiation process by which cells undergo a morphological switch from the epithelial polarized phenotype to the mesenchymal fibroblastoid phenotype, and involves loss of cell polarity, decreased cell-to-cell adhesion, and increased motility and capacity for migration [3]. EMT has been suggested to be an essential step in cancer cell dissemination and metastasis. During the process of tumor metastasis, which is often enabled by an EMT, disseminated cancer cells would seem to require self-renewal capability, in order to spawn macroscopic metastases. Recent work revealed that the process of EMT generates cells with stemlike properties in the mammary cell population [4]. The link between EMT and acquisition of stem cell-like properties by cancer cells may explain why EMT induces tumor progression. However, the mechanisms that induce and then maintain this mesenchymal/stem cell state remain unclear.

The acquisition of metastatic ability by tumor cells is considered a late event in the evolution of malignant tumors, in which the metastatic cell is presumed to arise progressively and step-wise to accumulate the additional mutations required for motility. Recently however, this paradigm has been challenged. New evidence suggests that malignant cells can disseminate at a much earlier stage than previously recognized in tumorigenesis [5], [6]. This suggests that an earlier trigger must be driving the development of the motile phenotype, enabling some of these cells to break free from the primary tumor, invade the microvasculature, travel and establish foci at distant sites. The formation of hybrids between cancer cells and normal bone marrow-derived cells within tumor-associated stroma has been advocated as a nonmutational mechanism that could contribute to aberrant gene expression patterns associated with highly malignant subpopulations [7]–[11].

Cell fusion is a fundamental process that occurs in both health and disease, in which two or more cells become one by merging their plasma membranes and rearranging their nuclear contents. The progeny of cell fusion are known as hybrids. Such fusion hybrids share the genetic and functional characteristics of both parent cells [7], [9], [12]. The genome of cancer cells might contribute tumorigenicity to the hybrids, whereas myeloid cells could contribute expression of mesenchymal genes and increased metastatic potential.

During the past decade, several distinct subsets of tumor-infiltrating myeloid cells have been described [13], among which mesenchymal stem cells (MSCs) have drawn attention for having a role in cancer progression [14]–[16]. MSCs are a small population of cells within the mesenchymal stromal cell compartment that have the capacity to self-renew and to differentiate into multiple cell lineages. MSCs infiltration is common in NSCLS [17]. In the majority of cases, tumor-infiltrating MSCs provide various functional aids to promote malignancy, ranging from structural support to creating a pre-metastatic environment [18], [19].

Taken together, these diverse lines of evidence suggest the hypothesis that fusogenic myeloid cell populations, such as MSCs, could facilitate the ability of tumor cells to acquire mesenchymal and stem cell-like properties by transferring distinct cellular capabilities during a physical fusion event with cancer cells. Therefore, the aim of this study was to identify whether hybrids formed by fusion between human lung cancer cells and bone marrow-derived MSCs could become a resource for mesenchymal/cancer stem-like cells.

## Results

### Spontaneous Formation of MSC–lung Cancer Hybrids

Cultured MSCs exhibited fibroblast-like cell morphology (Figure S1A), typical phenotypes and were positive for CD44, CD105 and negative for CD34 (Figure S1B), and have multipotency to differentiate into osteoblasts, adipocytes and chondrocytes after osteogenic, adipogenic and chondrogenic induction differentiation (Figure S1C-E).

**Figure 1:**
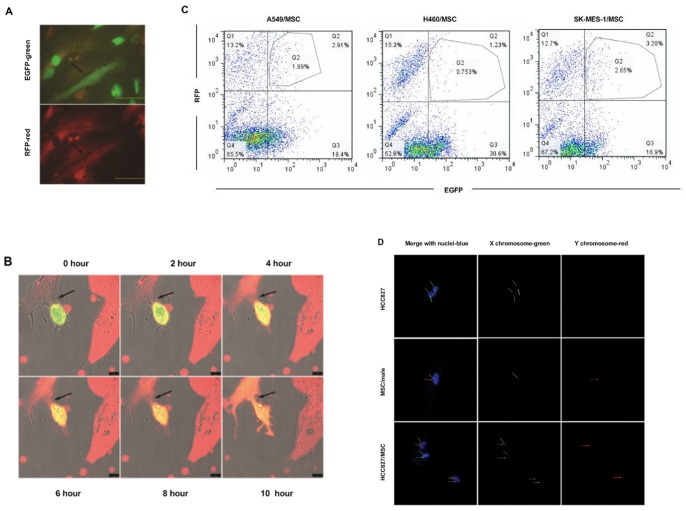
Spontaneous formation of heterotypic hybrids between lung cancer cells and MSCs. (**A**) Fluorescent micrographs of a co-culture of A549-EGFP (green) and MSC-RFP (red), showing formation of double-labeled hybrid cell with two nuclei and a fibroblastic shape (arrows). Scale bar, 50 µm. (**B**) Fluorescent micrographs, taken at 2-hourly intervals, of co-cultures of SK-MES-1-EGFP (green) with MSC-RFP (red), showing apparent involvement of cell fusion (arrows) and change in color in the interaction of lung cancer cells with MSCs. Scale bar, 10 µm. (**C**) Quantification of spontaneous hybridization between lung cancer cells (A549-EGFP, H460-EGFP or SK-MES-1-EGFP) and MSC-RFP in high-density co-culture. The number of hybrids was assessed by flow cytometric analysis and expressed as a percentage of the total cells. Data represent the mean ± SEM of three experiments. (**D**) FISH analysis of HCC827/MSC co-cultures. Male MSCs were cultured with female HCC827 lung cancer cells for 8 days and fixed. FISH (Spectrum red-Y chromosome and Spectrum green-X chromosome) was performed and nuclei were stained with DAPI (blue). Green arrows show X chromosomes and red arrows, Y chromosomes. Male MSCs expressed one Y chromosome and one X chromosome whereas female HCC827 cells expressed only X chromosomes. In HCC827/MSC hybrids: example of two cells, one harboring two nuclei and the other with one nucleus. These two cells each possess a Y chromosome indicating an MSC-derived cell. Scale bar, 25 µm.

We first evaluated whether spontaneously-formed heterotypic hybrids could be found in a co-culture experiment. The MSCs were fluorescently tagged by retroviral transduction with RFP-expressing vectors, and lung carcinoma cells were tagged by EGFP-expressing vectors. We co-cultured human MSCs with human A549, H460, or SK-MES-1 lung cancer cells. After 72 hours, cells expressing both EGFP and RFP were detected (Figure 1A). As cell fusion progressed, we observed MSCs gradually turning green and lung cancer cells gradually turning red, thus dually fluorescent cells, with distinct stromal morphology and emitting yellow fluorescence, emerged (Figure 1B). The presence of two or more nuclei was detected in most of these cells. The heterotypic hybrid population constituted 2.91±0.35%, 1.23±0.13% or 3.20±0.48% of the total cells in MSC-A549 co-culture, MSC-H460 co-culture or MSC-SK-MES-1 co-culture, respectively, as assessed by flow cytometry (Figure 1C). However, a control experiment showed that cell fusion was relatively non-detectable when co-culturing human MSCs with BEAS-2B (normal human bronchial epithelial cell line) or MRC-5 (normal human lung fibroblast cell line), respectively.

In order to confirm the role of fusion, gender-mismatched MSCs and HCC827 cells were co-cultured and heterotypic hybrids were evaluated by FISH of sex chromosomes. For these experiments, female HCC827 cells were rendered puromycin resistant by lentiviral transduction. The HCC827/MSC hybrids were selected by exposure to 1 µg/mL puromycin, which caused complete disappearance of male MSCs. After 5 days of selection, the appearance of male-derived hybrids with stromal morphology was detected. Some of the hybrids contained a single nucleus with one Y chromosome and several X chromosomes, indicating that the nucleus of a male MSC had fused with the nucleus of a female HCC827 cell. Some cells contained two nuclei, one with one Y chromosome and the other without a Y chromosome, indicating that the membrane of a male MSC had fused with the membrane of a female HCC827 cell without fusion of the two nuclei (Figure 1D). Other cells contained a single nucleus with one Y chromosome and one X chromosome, suggesting mitosis of fused multinucleated cells giving rise to MSC-derived mononucleated daughter cells (Figure S2A).

In order to confirm the occurrence of fusion *in vivo*, gender-mismatched MSCs and HCC827 cells were freshly mixed at the same ratio as that used in *in vitro* assays then xenografted by subcutaneous injection into NOD/SCID immunodeficient mice. Cell fusion between MSCs and HCC827 cells was evaluated by FISH of sex chromosomes. Five weeks after xenografting, spontaneously-formed male-derived tumorigenic hybrids were detected (Figure S2B). Collectively, these data indicate that spontaneously-formed MSC-lung cancer hybrids can be observed both *in vitro* and *in vivo*.

### Fusion between MSCs and Lung Cancer Cells Induces Morphologic Changes and Growth Inhibition

**Figure 2:**
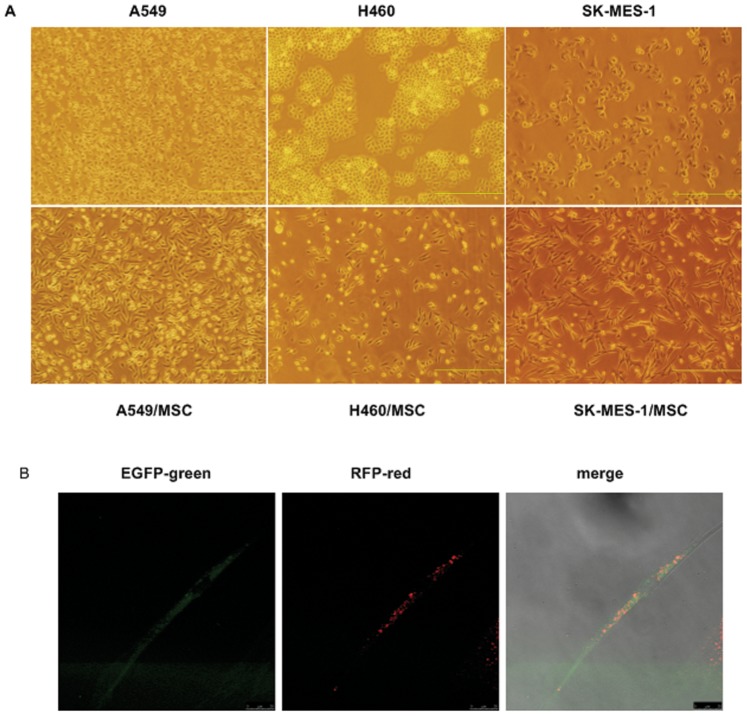
Fusion between lung cancer cells and MSCs induces morphological changes. (**A**) Phase-contrast images of heterotypic hybrids and respective parental lung cancer cells. After fusion with MSCs, lung cancer cells dispersed, lost their epithelial morphology and assumed a fibroblast-like appearance with elongated shape and front-to-back polarity. Scale bar, 500 µm. (**B**) Confocal laser microscopy imaging. A549/MSC hybrids assumed a fibroblast-like appearance and expressed both EGFP and RFP. Scale bar, 25 µm.

At 5 to 7 days after the end of puromycin treatment, we isolated puromycin-resistant fluorescent hybrids by fluorescence-activated cell sorting. After fusion with MSC-RFP, A549-EGFP cells lost their epithelial morphology. They became dispersed and assumed a fibroblast-like appearance with an elongated shape and front-to-back polarity. The same effect was observed with the H460-EGFP and SK-MES-1-EGFP cells (Figure 2A). These hybrids expressed both EGFP and RFP (Figure 2B), and two or more nuclei were detected in most of these cells. Our observations suggest that a transition from epithelial to mesenchymal–like morphological characteristics was induced by fusion.

Next, we tested the hypothesis that fusion with MSCs could influence cancer cell proliferation. In all cell lines tested (A549, H460 and SK-MES-1), fusion with MSCs induced growth inhibition. Hybrids began to grow slower than respective parental lung cancer cells from Day 2, and the difference of growth speed between the two groups was significant after Day 4 (Figure S3). These findings indicate that the proliferative potential of hybrids decreased significantly compared with respective parental lung cancer cells at each time point after Day 2.

### Fusion with MSCs Promotes a Shift from Epithelial to Mesenchymal Phenotype in Lung Cancer Cells

In view of the morphological changes of MSC-lung cancer hybrids, we next investigated whether they acquired a shift in expression from an epithelial to a mesenchymal repertoire. We used immunofluorescence and QRT-PCR to examine the expression and distribution of E-cadherin, pancytokeratins, vimentin, α-smooth muscle actin (α-SMA) and fibronectin in heterotypic hybrids and in their respective parental lung cancer cells. We also used QRT-PCR to examine the expression of EMT-inducing transcription factors such as Snail, Slug, Zeb1, Zeb2, Foxc2 and Twist genes.

**Figure 3:**
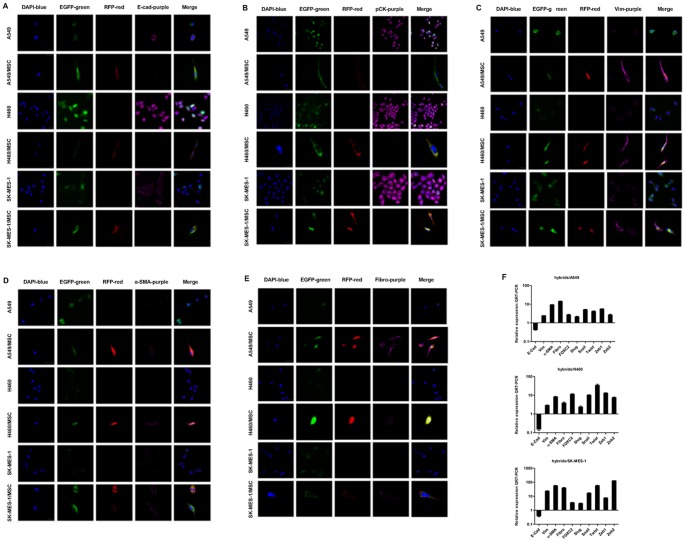
Expression of epithelial and mesenchymal markers in heterotypic hybrids and respective parental lung cancer cells. The MSCs and lung cancer cells were fluorescently tagged by retroviral transduction with RFP- and EGFP-expressing vectors, respectively. Spontaneously-formed heterotypic hybrids expressed both EGFP and RFP. Immunostaining was performed with primary antibodies against (**A**) E-cadherin (E-Cad), (**B**) pancytokeratin (pCK), (**C**) vimentin (Vim), (**D**) α-SMA, and (**E**) fibronectin (Fibro), and revealed using AlexaFluor 647-labelled secondary antibodies (purple); nuclei were stained with DAPI (blue). Scale bar, 25 µm. (**F**) The expression levels of the mRNAs encoding E-cadherin, vimentin, α-SMA, fibronectin, Foxc2, Slug, Snail, Twist, Zeb1 and Zeb2 in spontaneously formed A549/MSC, H460/MSC, SK-MES-1/MSC heterotypic hybrids relative to respective parental lung cancer cells as determined by QRT-PCR. GAPDH mRNA was used to normalize the variability in template loading. The data are reported as mean ± SEM.

Immunofluorescence staining showed that, compared with parental lung cancer cells, MSC-lung cancer hybrids exhibited a strong reduction in protein expression of E-cadherin and pancytokeratins as well as significantly increased expression of vimentin, α-SMA and fibronectin in all three NSCLC cell lines (Figures 3A–E).

QRT-PCR analyses revealed that, relative to expression in parental lung cancer cells, the level of E-cadherin mRNA in hybrids was greatly reduced, while expression of mRNAs encoding mesenchymal markers was markedly increased (Figure 3F). There was also a considerable increase in the expression of EMT-inducing transcription factors (Figure 3F).

In order to determine the capacity of lung cancer cells to acquire mesenchymal characteristics without fusion events, EGFP-expressing lung cancer cells and RFP-expressing MSCs were plated in the top and bottom wells of a 0.4 µm-cell culture insert. After indirect co-culture for 8 days, lung cancer cells exhibited similar expression of E-cadherin, vimentin, α-SMA and fibronectin in all three NSCLC cell lines compared with parental lung cancer cells that were cultured alone (data not shown). These results demonstrated that MSC-lung cancer hybrids expressed markers associated with cells that have undergone an EMT.

### Hybrids Acquire Increased Motility and Invasiveness

**Figure 4:**
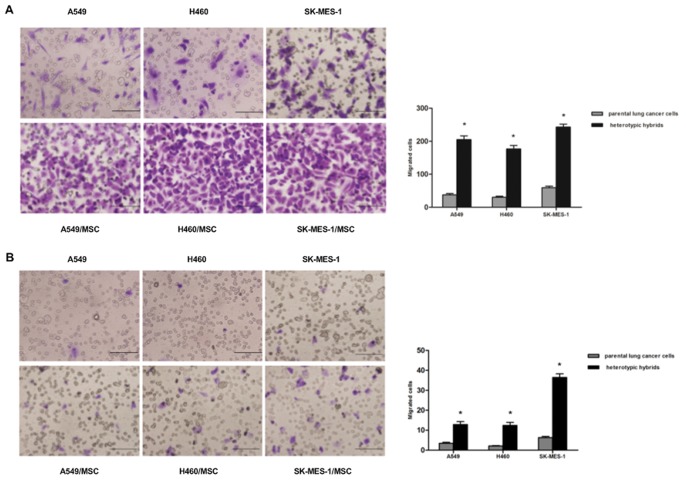
Migration and invasion assays of heterotypic hybrids and respective parental lung cancer cells. The migratory and invasive potential of the cells was determined by counting the number of cells that had migrated to the lower surface of the filter in 12 randomly selected microscopic fields per insert. (**A**) Migration assays: microscopic images and quantification. (**B**) Invasion assays: microscopy images and quantification. The data are reported as mean ± SEM of three independent experiments. Asterisks depict statistically-significant differences between the heterotypic hybrids and respective parental lung cancer cells (*P<0.001). Scale bar, 50 µm.

To further investigate another functional hallmark of the mesenchymal/stem cell state, we conducted *in vitro* motility assays. In migration assays, A549/MSC hybrids were more migratory (5.5-fold) compared to parental A549 cells, H460/MSC hybrids were more migratory (5.9-fold) compared to parental H460 cells, and SK-MES-1/MSC hybrids were more migratory (4.1-fold) compared to parental SK-MES-1 cells (Figure 4A). In invasion assays, A549/MSC hybrids were more invasive (3.8-fold) compared to parent A549 cells, H460/MSC hybrids were more invasive (5.9-fold) compared to parent H460 cells, and SK-MES-1/MSC hybrids were more invasive (5.8-fold) compared to parent SK-MES-1 cells (Figure 4B). Taken together, our observations indicate that spontaneously-formed hybrids between lung cancer cells and MSCs represent a subpopulation enriched for motile cells.

### Gene Expression Profiling of Stemness Markers in Hybrids

**Figure 5:**
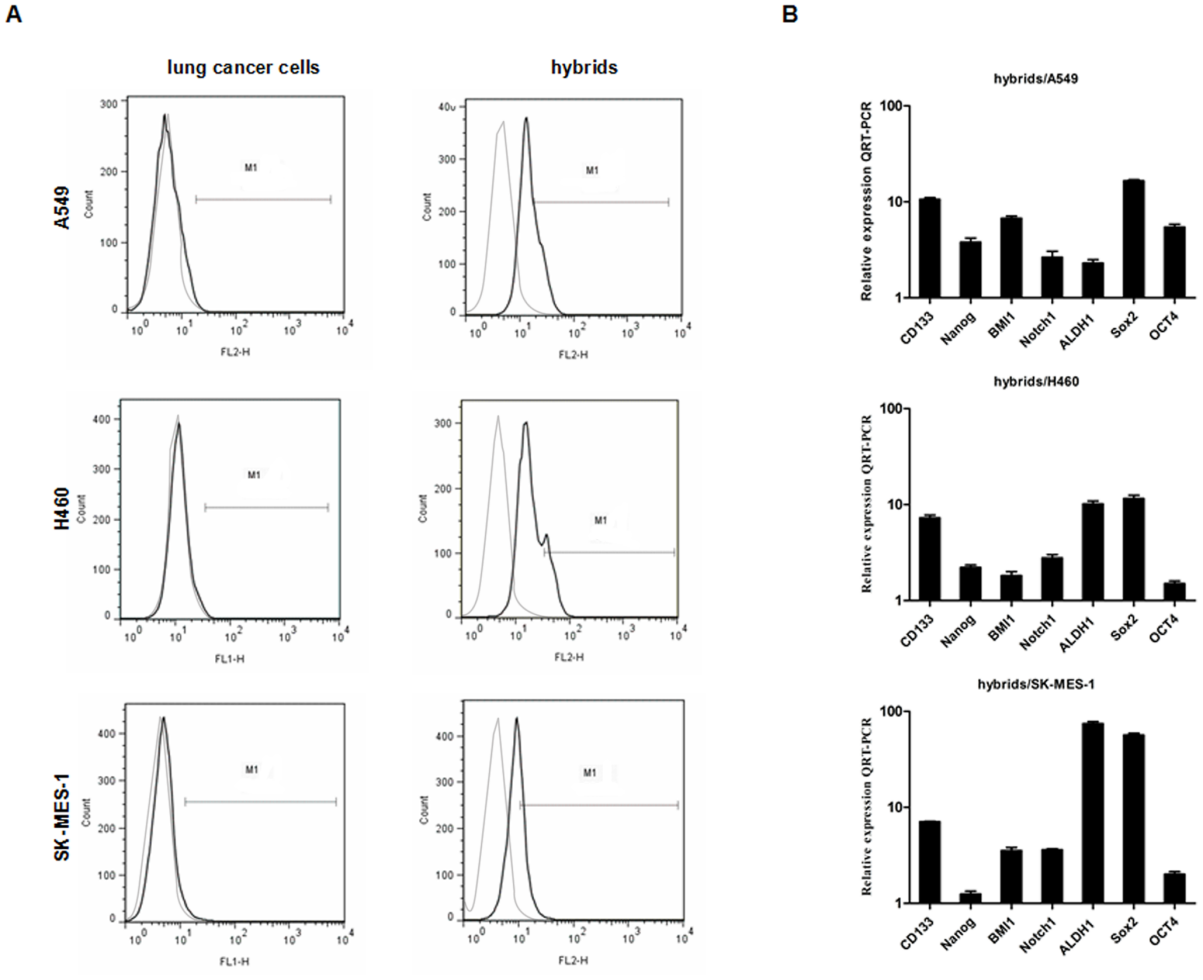
Expression of stemness markers in heterotypic hybrids and respective parental lung cancer cells. (**A**) Flow cytometry analysis of CD133 expression in heterotypic hybrids and respective parental lung cancer cells. Percentages of CD133^+^ cells are indicated in control antibody and specific antibody-stained cells. Grey and black line indicated negative control and sample, respectively. (**B**) The expression levels of the mRNAs encoding CD133, Nanog, BMI1, Notch1, ALDH1, Sox2 and OCT4 in spontaneously-formed A549/MSC, H460/MSC and SK-MES-1/MSC heterotypic hybrids relative to respective parental lung cancer cells as determined by QRT-PCR. GAPDH mRNA was used to normalize the variability in template loading. The data are reported as mean ± SEM.

The stem cell marker prominin-1 (CD133), a pentaspan membrane protein, may not be the only marker, but it remains the most widely reported marker of cancer stem cells (CSCs) of lung cancer, and has been validated by different groups [20]–[22]. We further assessed the expression of CD133 on MSC-lung cancer hybrids using flow cytometry and QRT-PCR. Flow cytometry analyses showed that in parental A549 cells, the percentage expressing CD133 was about 0.94% of the total cell population. After fusion with MSCs, the expression of CD133 increased 30-fold. Additionally, CD133 expression increased in hybrids compared with their respective parental H460 and SK-MES-1 cell lines (Figure 5A). QRT-PCR analyses also revealed that, relative to the expression in parental lung cancer cells, the level of CD133 mRNA in hybrids was significantly increased (Figure 5B). These data provide further evidence that spontaneously-formed tumorigenic hybrids between lung cancer cells and MSCs acquire over-expression of stem cell markers accompanied by passage through an EMT.

To investigate the genes responsible for regulating and maintaining the stem cell phenotype of A549/MSC hybrids, we performed QRT-PCR for OCT4, Nanog, BMI1, Notch1, ALDH1 and Sox2. Interestingly, the transcription factors OCT4, Nanog, BMI1, Notch1, ALDH1 and Sox2, known to be sufficient to reprogram mouse or human somatic cells to undifferentiated, pluripotent stem cells, were found to be significantly increased in hybrids compared to parental lung cancer cells, indicating their stemness phenotype (Figure 5B). These findings indicate that self-renewal genes are over-expressed in spontaneously-formed hybrids between lung cancer cells and MSCs.

### Ability to form Tumor Spheres is Increased in Hybrids

**Figure 6:**
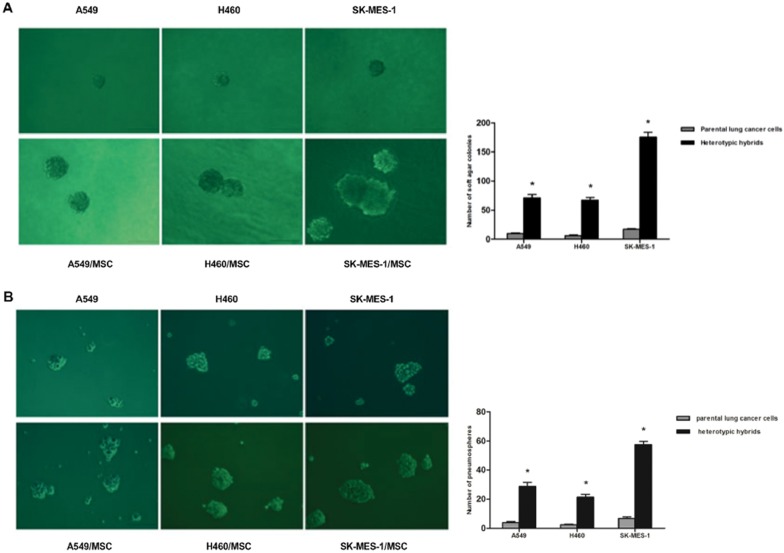
Tumor sphere formation ability of heterotypic hybrids and respective parental lung cancer cells. (**A**) Soft agar assays: Single cells (1×10^3^ per well) were plated into soft agar in 6-well plates in triplicate. Microscopic images and quantification of heterotypic hybrids and respective parental A549, H460 or SK-MES-1 cells. The data are reported as mean ± SEM. (**B**) Pneumosphere assay: Microscopic images and quantification of heterotypic hybrids and respective parental A549, H460 or SK-MES-1 cells; n = 12. The data are reported as mean ± SEM. Asterisks depict statistically-significant differences between the heterotypic hybrids and respective parental lung cancer cells (*P<0.001). Scale bar, 100 µm.

The soft agar assay serves as an *in vitro* surrogate measure of tumorigenicity [23], while the pneumosphere assay measures anchorage-independent proliferation at clonal density *in vitro* and has been associated with the presence of stem cell populations [24]. We therefore subjected these cells to both soft agar and pneumosphere assays. Interestingly, we observed that the heterotypic hybrids formed at least 7.5-fold more colonies in soft agar suspension culture than did the control parental lung cancer cells (Figure 6A and Figure S4A). Equally important, hybrid cells showed significantly increased ability to form pneumospheres compared to their parental lung cancer cells, forming at least 7.3-fold more pneumospheres than their parental cells (Figure 6B). The pneumosphere size of hybrid cells was also significantly greater than that of parental lung cancer cells. By dissociating the initially-formed mammospheres and reintroducing these cells into secondary mammosphere cultures, we observed a modest increase in sphere formation by hybrids (Figure S4B). Based on this assay, we concluded that spontaneously-formed tumorigenic hybrids between lung cancer cells and MSCs acquire yet another attribute of CSCs.

### Heterotypic Hybrids Exhibit enhanced Tumorigenicity and Reproduce the Human Tumor *in vivo*

**Figure 7:**
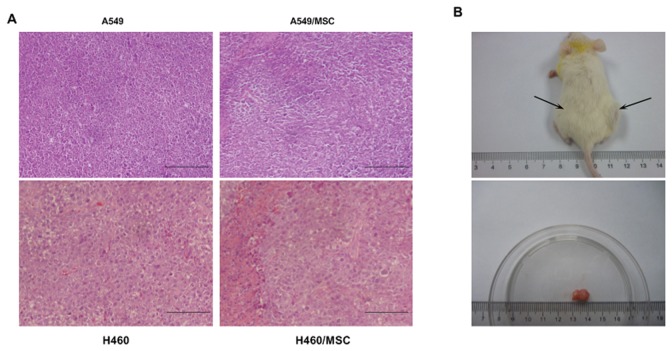
Tumorigenic potential of MSC-lung cancer hybrids. (**A**) MSC-lung cancer hybrids are tumorigenic and regenerate similar tumors to their respective parental lung cancer cells in NOD/SCID mice. Hematoxylin and eosin (H&E) staining performed on tumor specimens derived from parental tumor or from tumors generated by subcutaneous injection of MSC-lung cancer hybrids in NOD/SCID mice (xenograft). Data are representative of three independent experiments. (**B**) ***Upper panel:*** Tumorigenic potential of 1×10^3^ A549/MSC compared with 1×10^4^ parental A549 cells. Cells were simultaneously injected into the right (A549/MSC) and left flank (A549) of the same mouse, and the mouse photograph was taken 12 weeks after injection. ***Lower panel:*** Gross pathology of tumor generated by subcutaneous injection of A549/MSC hybrids in the upper panel. Data are representative of three independent experiments. Scale bar, 100 µm.

In order to test whether fusion with MSCs succeeds in altering the tumor-initiating frequency of lung cancer cells, we injected A549/MSC hybrids, H460/MSC hybrids as well as SK-MES-1/MSC hybrids and respective parental lung cancer cells into NOD/SCID mice. As reported in Table 1, we found that injection of as few as 10^4^ hybrids consistently resulted in the growth of tumor xenografts with morphological features closely resembling the tumor induced by the respective parental lung cancer cells, as shown by hematoxylin and eosin staining (Figure 7A). The volume of tumor generated by hybrids was significantly larger than that resulting from parental cells (Figure 7B). These results confirmed that spontaneously-formed tumorigenic hybrids between lung cancer cells and MSCs represent a subpopulation enriched for lung cancer–initiating cells.

**Table 1:** Tumorigenic Potential of MSC-lung Carcinoma Hybrids.

	Tumor incidence/number of injections					
Number of cells injected	A549	A549/MSC	H460	H460/MSC	SK-MES-1	SK-MES-1/MSC
1×10^6^	8/10	10/10	9/10	10/10	8/10	10/10
1×10^5^	3/10	9/10*	3/10	8/10*	4/10	8/10*
1×10^4^	0/10	5/10*	0/10	4/10*	0/10	6/10*
1×10^3^	0/10	2/10*	0/10	2/10*	0/10	3/10*

Note: Data are given as number/total (percentage). Control MSCs implanted alone into 10 mice (inoculum, 1×10^6^ cells/mouse) were nontumorigenic.

*Statistically significant difference by two-sided Fisher’s exact test with respect to the rate of tumor incidence by parental lung cancer cells (P<0.001).

### Hybrids Cells Reacquire Epithelial-like Morphological Characteristics *in vitro* and *in vivo* Upon Differentiation

**Figure 8:**
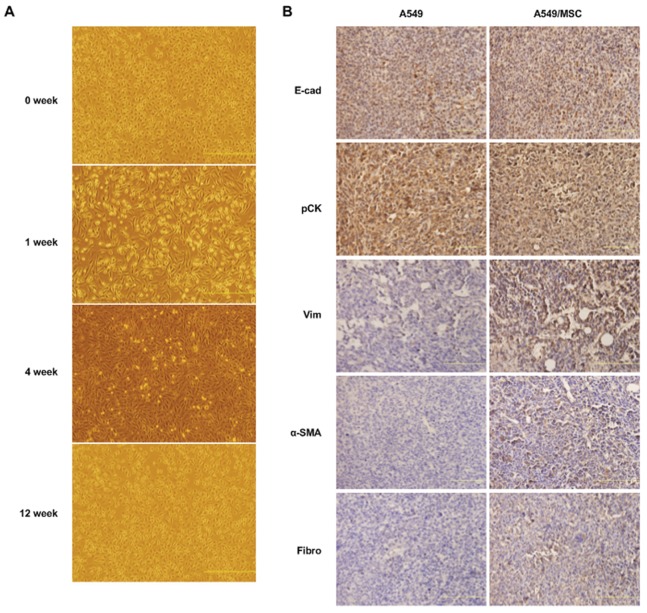
Reversion of A549/MSC hybrids from fibroblast-like to epithelial morphological characteristics *in* *vitro* and *in vivo*. (**A**) Images, at successive time points, showing reversion of the fibroblastic morphological characteristics of A549/MSC hybrids to an epithelial, A549-like appearance in culture. Scale bars: 500 µm. (**B**) Immunohistochemical staining for the indicated antigens performed on tumor specimens derived from parental A549 tumor or from tumors generated by subcutaneous injection of A549/MSC hybrids in NOD/SCID mice. Explants of tumor xenografts of A549/MSC hybrids and parental A549 cells displayed similar lung cancer-like morphological characteristics. Scale bar, 100 µm.

After 3 months in culture, a generalized transition from mesenchymal to epithelial-like morphological characteristics was observed for MSC-lung cancer hybrids (Figure 8A). A similar morphological change was also observed during growth of tumor xenografts. The cell shape of hybrid xenografts was much more like that of the parental lung cancer cells in appearance. Strong membranous expression of pancytokeratins and E-cadherin in hybrid xenografts, comparable with the expression in parental lung cancer xenografts, and cell morphology were suggestive of the epithelial nature of the tumors, whereas enhanced expression of vimentin, α-SMA and fibronectin suggested an MSC contribution to the hybrid phenotype (Figure 8B). These data suggest the instability of the hybrids. As time goes on, a reversal of the mixed phenotype and the reverse process termed mesenchymal-epithelial transition (MET) takes place.

## Discussion

The findings presented here describe an unexpected convergence of EMT and stem cell properties in spontaneously-formed tumorigenic hybrids between lung cancer cells and bone marrow-derived MSCs. The epithelial-mesenchymal transition (EMT) has been described over the past decade as a critical normal process during development and wound healing, but recently properties of EMT have been implicated in human pathology, including cancer metastasis [25]. Independent of these findings, studies have described evidence of stem cells in a variety of cancers. Mani *et al.* first found a link between these two sets of phenomena in breast tumor cells in 2008 [4]. However no research has yet revealed the mechanisms behind them, or identified the source of the cells with both EMT and stem cell properties. In this study, we demonstrate for the first time that lung cancer cells can acquire mesenchymal characteristics and stem cell properties through a fusion mechanism with MSCs. The discovery of spontaneously-formed tumorigenic hybrids between lung cancer cells and bone marrow-derived MSCs with many of the properties of both EMT and self-renewing stem cells holds the promise of resolving a major problem in cancer biology. During the process of tumor metastasis, EMT properties may permit dissemination of tumor cells from the primary tumor into the surrounding stroma and thus to distant sites [26]. However, if the vast majority of cells that are spread lack self-renewal capability, their ability to found macroscopic metastases is compromised from the outset because of their limited proliferative potential. This mystery may be solved, at least in part, by the present findings, since the spontaneously-formed hybrids between cancer cells and MSCs obtain both EMT and stem cell-like properties, development of which enables cancer cells to disseminate from a primary tumor and to form colonies at a distance.

Recently, the link between cancer progression and infiltration with myeloid cells has been recognized in a variety of cancers [13]. Infiltration with myeloid cells is usually associated with less favorable clinical outcomes [27]–[29]. Inflammation is an important constituent of the local environment of tumors [30]. Chemokines and cytokines are crucial elements, which contribute to cancer-related inflammation [31], [32]. Attracted by chemokines, a variety of MSCs from the bone marrow are recruited to tumor sites, and play a role in facilitating lung cancer progression [17]. Recent work indicates that chronic inflammation can promote cell fusion [33]–[35]. The present study demonstrates for the first time that MSCs can promote EMT and dissemination by means of fusion with lung cancer cells. It also provides a mechanistic explanation for the long-recognized link between inflammation and cancer progression.

The present study demonstrates that cell fusion between MSCs and lung cancer cells results in a subpopulation exhibiting mesenchymal morphological characteristics and cytogenetic abnormalities, along with invasive features and self-renewal capability. Clearly, the genome of the lung cancer cells contributed tumorigenicity to the hybrids, whereas MSCs contributed expression of mesenchymal genes and increased metastatic potential. Some of the hybrids may also gain the capacity to self-renew and to differentiate into multiple cell lineages from the bone marrow-derived MSCs. Interestingly, in a previous study, spontaneously-formed tumorigenic hybrids between MSCs and breast cancer cells had a mesenchymal-like appearance and mixed expression of mesenchymal and breast carcinoma genes [9]. Our data are consistent with previous reports that spontaneously-fused tumorigenic hybrids between MSCs and cancer cells can subsequently adopt the phenotype of the recipient cells [9], [12]. The *in vivo* tumorigenic assay confirmed their tumorigenic properties, while increased expression of vimentin, α-smooth muscle actin (α-SMA) and fibronectin, and overexpression of transcription-regulating genes, such as Slug, Snail, Zeb1, Zeb2, Foxc2 and Twist, indicate that hybrids may acquire mesenchymal properties from MSCs. Furthermore, overexpression of self-renewal genes such as Bmi1, ALDH1, and Oct-4 indicates that lung cancer cells may acquire stem cell-like properties from MSCs by fusion.

Cogle *et al.* demonstrated no evidence of a fusion karyotype between epithelial cells and bone marrow cells using confocal microscopy and XY enumeration since donor-derived (Y chromosome positive) cells detected by FISH contained only one X chromosomes [36]. The paradoxical results mentioned above might due to the instability of hybrids. Within 3 months, both *in vitro* and *in vivo*, the morphological characteristics of hybrid cells changed. This indicates that hybrids are unstable. Cells that arise as fusion products may undergo “reduction division,” and DNA ploidy decreased within 60 days [9]. Since the tumor-forming time in our murine model was about 5 weeks, we could detect hybrids containing male specific chromosomes but more than two X chromosomes. In agreement with our data, Rappa *et al*. also observed the transition from mesenchymal to epithelial-like morphological characteristics in hybrids generated by fusion between MSCs and breast cancer cells [9]. For a long time, the histopathological similarity of metastatic lesions to their primary tumor counterparts argued against the EMT theory. Our study provides a mechanistic explanation for this apparent contradiction. Strong membranous expression of pancytokeratins and E-cadherin was observed in xenografts formed by heterotypic hybrids and cell morphology was suggestive of the epithelial transition of hybrids. The instability of the hybrids also provides a possible mechanistic explanation for a subsequent step, mesenchymal-epithelial transition (MET).

The connections described here appear to hold a number of implications for the biology of epithelial cancer cells. Fusion events between MSCs and lung cancer cells may result in the transient induction of an EMT in large populations of cancer cells and at the same time may make possible the generation of relatively unlimited numbers of cancer stem cells, whose biology may then be studied with far greater facility.

In conclusion, our data show that fusion between MSCs and lung cancer cells generates a new hybrid offspring with both EMT and stem cell-like properties. The generation of unlimited numbers of hybrids with both EMT and stem cell-like properties by use of cell fusion techniques could contribute significantly to the understanding of lung cancer biology. Where such fusions spontaneously form compatible genetic re-assortments, lung cancer subpopulations with increased malignancy and prometastatic traits are created.

## Materials and Methods

### Ethics Statement and Cell Culture

Approval was obtained from the Institutional Ethical Board and the local Central Animal Facility Committee of the Third Military Medical University before initiation of the study. This study was conducted according to institutional guidelines under an approved protocol. BEAS-2B, MRC-5, A549, H460, SK-MES-1 and HCC827 cell lines were obtained from the American Type Culture Collection (ATCC, Manassas, VA, USA) and cultured according to ATCC’s protocols. Human bone marrow-derived MSCs/red fluorescent protein (RFP) were obtained commercially (Cyagen Biosciences, Guangzhou, CHN) and cultured in MSCs growth medium (Cyagen Biosciences, Guangzhou, CHN) at 37°C under 5% CO_2_ and 90% humidity. The medium was changed every three days. The third-sixth passage MSCs were used for experiments.

### Co-culture

Direct co-culture experiments with cancer cells and MSCs were performed in MSCs medium. To form a stromal cell monolayer, 5×10^5^ red fluorescent-labeled MSCs in 2 mL medium were plated into each well of a 6-well plate (Corning Inc., Corning, NY, USA) and allowed to grow to 90% confluence. The medium was removed and 5×10^5^ green fluorescent-labeled lung cancer cells (A549, H460, or SK-MES-1) in 2 mL fresh medium were overlaid onto the monolayer. A 0.4 µm cell culture insert system was used for the indirect co-culture assay (BD Biosciences, San Jose, CA, USA). The co-culture medium was changed every three days.

### Migration and Invasion Assays

For *in vitro* migration assays, 2×10^4^ cells in serum-free α-MEM were seeded into 24-well cell culture inserts with 8 µm pores (BD Biosciences). For *in vitro* invasion assays, 1×10^5^ cells in serum-free α-MEM were seeded into Biocoat Matrigel Invasion Chambers containing BD Falcon Cell Culture Inserts with 8 µm pores that had been coated with Matrigel Matrix (BD Biosciences). The lower chambers were filled with α-MEM containing 10% fetal bovine serum. After a 24-hour incubation at 37°C, the cells on the upper side of the membrane were gently removed with wet cotton swabs. The cells on the lower surface of the membranes were fixed with 4% paraformaldehyde for 10 minutes and then stained with crystal violet hydrate solution (Sigma-Aldrich, St Louis, MO, USA). The migratory and invasive potential of the cells in both migration and invasion assays was determined by counting the number of cells that had migrated to the lower surface of the filter in 12 different areas under an inverted light microscope (Nikon TS 100, Milan, Italy). Each assay was performed in triplicate in three separate experiments.

### Proliferation Assay

Cell proliferation was assessed at 24-hour intervals for eight days using the CCK-8 assay according to the manufacturer’s instructions. Briefly, Cells were seeded into a 96-well plate at a density of 1×10^3^ cells/well and cultured in α-MEM supplemented with 10% FBS. The plate was incubated in a humidified incubator (37°C, 5% CO_2_) for 1–8 days. Four hours before measuring the absorbance, 10 µl of the CCK-8 solution (Sigma, St. Louis, MO, USA) was added into each well. Absorbance was measured at a wave length of 450 nm using a microplate reader (Biotek Instrument, Winooski, USA). The experiments were repeated three times.

### Soft Agar Assay

Cells were suspended in serum-free α-MEM at a density of 1×10^3^ and 1×10^5^ cells per well in 6-well plates (Corning) and plated in soft agar, in triplicate. The test was performed using 0.7% and 0.3% agar in α-MEM as the base and top layers, respectively. Cells were incubated for 21 days at 37°C in a humidified atmosphere of 5% CO_2_ in air. At the end of the incubation period, colony cultures were photographed, and colonies with diameters larger than 50 µm were counted.

### Culture of Pneumospheres

Cells were suspended in serum-free α-MEM supplemented with 20 ng/mL EGF, 10 ng/mL bFGF, and B27 (BD Biosciences) and 1×10^3^ cells/well were seeded into 24-well ultra-low adhesion plates (Corning). For secondary sphere formation, primary spheres were dissociated by trypsinization and replated at 1×10^3^ cells/well. The medium was changed daily along with growth factor supplementation until cells started to grow forming floating aggregates. The pneumospheres were cultured for 5–7 days, and then pneumospheres with diameter larger than 50 µm were counted.

### Cell Labeling

Lung cancer cells transduced with lentiviral particles containing vectors encoding EGFP (Invitrogen, Carlsbad, CA, USA) at multiplicities of infection ranging from 1 to 40. Lung cancer cells were also tagged and rendered puromycin resistant by lentiviral transduction. Twenty-four hours after transduction, transduction efficiency was measured by flow cytometry and positive cells were sorted to obtain a homogeneous population. For staining with fluorescent chemical compounds, the PKH26 Red Fluorescent Cell Linker Kit and PKH67 Green Fluorescent Cell Linker Kit (Sigma) were used according to the manufacturer’s recommendations. Briefly, cells were harvested and resuspended in 1 mL of labeling solution (PKH26 or PKH67) for the time indicated in the manufacturer’s instructions. Cells were washed three times in culture medium before use.

### Flow Cytometry Analysis and Cell Sorting of Heterotypic Hybrids

For flow cytometry, cells were washed and incubated with the appropriate dilution of isotype control or specific antibody. Antibody used was FITC-conjugated anti-CD133/2 from Miltenyi Biotec (Bergisch Gladbach, Germany). Staining was performed in the dark at 4°C for the time recommended by the manufacturer. After incubation, cells were washed before analysis using a FACScan flow cytometer (BD Biosciences). To select heterotypic hybrids, co-cultured MSCs and lung cancer cells were exposed to 1 µg/mL puromycin, which caused complete disappearance of MSCs. After 2 weeks of selection, hybrids double stained with EGFP and RFP were selected in a BD FACS Aria flow cytometer (BD Biosciences).

### Immunofluorescence Analysis

For immunofluorescence studies, cells were grown on poly-D-lysine-coated glass coverslips, fixed with 4% paraformaldehyde for 30 min at room temperature and permeabilized with 0.25% Triton X-100/PBS for 10 min at room temperature before incubation with the specific or control antibody. Primary antibodies against E-cadherin, pancytokeratin, vimentin, α-SMA and fibronectin were all mouse anti-human (all from Santa Cruz Biotechnology Inc., Santa Cruz, CA, USA), and were diluted at 1∶200. The secondary antibody, Alexa-647-labelled goat anti-mouse (Abcam, Bristol, UK, USA) diluted 1∶200 in PBS, was incubated for 60 min, and DAPI (Sigma), used as a nuclear stain was incubated for 10 min at room temperature. Cells were then washed twice and observed under a confocal microscope (Leica, Wetzlar, Germany). Fluorescence *In Situ* Hybridization (FISH) experiments were performed using probes against the α-satellite centromeric region of the X chromosome and the α-satellite centromeric region of the Y chromosome, according to the manufacturer’s instructions (Kreatech, Amsterdam, The Netherlands). Coverslips were washed and observed under a confocal microscope (Leica).

### Immunohistochemistry on Tumor Sections

Sections (4 µm thick) were incubated overnight at 4°C with the following antibodies: rabbit polyclonal anti-E-cadherin (Abcam), anti-pancytokeratin (Santa Cruz Biotechnology), anti-vimentin (Santa Cruz Biotechnology), anti-fibronectin (Abcam), and anti-α-SMA (Santa Cruz Biotechnology). The reaction was performed using PV-9000 Polymer Detection Systems (GBI, Mukilteo, WA, USA) and DAB substrate chromogen (GBI), followed by counterstaining with hematoxylin.

### Generation of Subcutaneous Lung Cancer Xenografts in SCID Mice

For mouse xenografts, cells were mechanically dissociated to obtain single cell suspensions, diluted in growth factor-containing medium and mixed with matrigel before subcutaneous injection into four-week-old female SCID mice. Serial dilutions of cells (down to as low as 1×10^3^ cells) were injected to evaluate the tumorigenic activity of hybrid cells. Mice were monitored to check for the appearance of signs of disease, such as subcutaneous tumors or weight loss due to potential tumor growth at internal sites. Mice were sacrificed after 12 weeks or when tumors reached a diameter >1 cm. and tumor tissue was collected, fixed in buffered formalin and subsequently analyzed by immunohistochemistry.

### Quantitative Real-time PCR

Total RNA was extracted using TRIzol Reagent (Invitrogen) according to the manufacturer’s protocol. cDNA was obtained from 1 ug of total RNA, using reverse transcriptase and random primers (Invitrogen) according to the manufacturer’s instructions. cDNAs (1 mL for each sample) were amplified by PCR. Primer sequences are listed (Table S1). GAPDH was amplified as an internal control. Thermal cycling parameters were: 95°C for 2 min, 35 cycles of 95°C for 30 sec, 52–60°C (depending on the Tm of each individual set of primers) for 1 min and 72°C for 30 sec. QRT-PCR was performed using SYBR Green PCR Master Mix (Invitrogen) using a CFX384 Touch™ instrument (Bio-Rad, Hercules, CA, USA). The expression level for each gene was normalized to that of the GAPDH housekeeping gene in the same sample.

### Statistical Analysis

All data are presented as mean ± SEM except where stated. When two groups were compared, a two-tailed Student’s t test was used to compare them. *P*<0.05 was considered statistically significant.

## Supporting Information

Figure S1 Characterization of MSCs. **(A)** Cultured MSCs exhibited fibroblast-like cell morphology. **(B)** Expression of CD44, CD105 and CD34 on MSC-lung cancer hybrids using flow cytometry. **(C)** Osteoblasts differention. **(D)** Adipocytes differention. **(E)** Chondrocytes differentiation. Scale bar, 25 µm.
(TIF)

Figure S2 FISH analysis of HCC827/MSC hybrids. **(A)** Male MSCs were cultured with female HCC827 lung cancer cells for 8 days and fixed. FISH (Spectrum red-Y chromosome and Spectrum green-X chromosome) was performed and nuclei were stained with DAPI (blue). Green arrows show X chromosomes and red arrows, Y chromosomes. Upper pictures show an HCC827/MSC hybrid harboring one nucleus with one Y chromosome and two X chromosomes; Lower pictures show an HCC827/MSC hybrid harboring one nucleus with one Y chromosome and one X chromosome. **(B)** Spontaneously-formed male-derived tumorigenic hybrids were detected *in vivo* by FISH. Tumor specimens were derived from tumors generated by subcutaneous injection of mixed MSCs and HCC827 cells (above) or from parental tumor (below) in NOD/SCID mice. Scale bar, 25 µm.
(TIF)

Figure S3 Growth curves of hybrids and respective parental lung cancer cells. A CCK-8 assay was performed and the absorbance were detected at 450 nm. **(A)** Growth curves of A549/MSC hybrids and A549. **(B)** Growth curves of H460/MSC hybrids and H460. **(C)** Growth curves of SK-MES-1/MSC hybrids and SK-MES-1. The data are reported as mean ± SEM of three independent experiments performed in triplicate. Asterisks depict statistically-significant differences between the heterotypic hybrids and respective parental lung cancer cells (*P<0.01).
(TIF)

Figure S4 Tumor sphere formation ability of heterotypic hybrids and respective parental lung cancer cells. **(A)** Soft agar assays: Single cells (1×10^5^ per well) were plated into soft agar in 6-well plates in triplicate. Quantification of heterotypic hybrids and respective parental A549, H460 or SK-MES-1 cells. **(B)** Secondary mammosphere quantification of heterotypic hybrids and respective parental A549, H460 or SK-MES-1 cells. Primary spheres were dispersed by trypsinization and replated at 1×10^3^ cells/well. The pneumospheres were cultured for 5–7 days, then pneumospheres with diameter larger than 50 µm were counted. n = 12. Data are reported as mean ± SEM. Asterisks indicate statistically-significant differences between the heterotypic hybrids and their respective parental non-small-cell lung cancer cells (*P<0.001).
(TIF)

Table S1 Forward and reverse primer sequences using real-time PCR.
(DOC)

## References

[1] Guo S, Reddy CA, Chao ST, Suh JH, Barnett GH, et al (2012) Impact of non-small cell lung cancer histology on survival predicted from the graded prognostic assessment for patients with brain metastases. Lung Cancer 77: 389–393. 22542706 10.1016/j.lungcan.2012.03.028

[2] Martínez-Moreno P, Nieto-Cerón S, Torres-Lanzas J, Ruiz-Espejo F, Tovar-Zapata I, et al (2006) Cholinesterase activity of human lung tumours varies according to their histological classification. Carcinogenesis 27: 429–436. 16272577 10.1093/carcin/bgi250

[3] Thiery JP, Acloque H, Huang RY, Nieto MA (2009) Epithelial-mesenchymal transitions in development and disease. Cell 139: 871–890. 19945376 10.1016/j.cell.2009.11.007

[4] Mani SA, Guo W, Liao MJ, Eaton EN, Ayyanan A, et al (2008) The epithelial-mesenchymal transition generates cells with properties of stem cells. Cell 133: 704–715. 18485877 10.1016/j.cell.2008.03.027 PMC2728032

[5] Bernards R, Weinberg RA (2002) A progression puzzle. Nature 418: 823. 12192390 10.1038/418823a

[6] Coghlin C, Murray GI (2010) Current and emerging concepts in tumour metastasis. J Pathol 222: 1–15. 20681009 10.1002/path.2727

[7] Powell AE, Anderson EC, Davies PS, Silk AD, Pelz C, et al (2011) Fusion between Intestinal epithelial cells and macrophages in a cancer context results in nuclear reprogramming. Cancer Res 71: 1497–1505. 21303980 10.1158/0008-5472.CAN-10-3223 PMC3079548

[8] Ding J, Jin W, Chen C, Shao Z, Wu J (2012) Tumor associated macrophage×cancer cell hybrids may acquire cancer stem cell properties in breast cancer. PLoS One 7: e41942. 22848668 10.1371/journal.pone.0041942 PMC3405038

[9] Rappa G, Mercapide J, Lorico A (2012) Spontaneous formation of tumorigenic hybrids between breast cancer and multipotent stromal cells is a source of tumor heterogeneity. Am J Pathol 180: 2504–2515. 22542847 10.1016/j.ajpath.2012.02.020 PMC3378856

[10] Wang R, Sun X, Wang CY, Hu P, Chu CY, et al (2012) Spontaneous cancer-stromal cell fusion as a mechanism of prostate cancer androgen-independent progression. PLoS One 7: e42653. 22880071 10.1371/journal.pone.0042653 PMC3411834

[11] Pawelek JM, Chakraborty AK (2008) Fusion of tumour cells with bone marrow-derived cells: a unifying explanation for metastasis. Nat Rev Cancer 8: 377–386. 18385683 10.1038/nrc2371

[12] Dittmar T, Schwitalla S, Seidel J, Haverkampf S, Reith G, et al (2011) Characterization of hybrid cells derived from spontaneous fusion events between breast epithelial cells exhibiting stem-like characteristics and breast cancer cells. Clin Exp Metastasis 28: 75–90. 20981475 10.1007/s10585-010-9359-3

[13] Bianchi G, Borgonovo G, Pistoia V, Raffaghello L (2011) Immunosuppressive cells and tumour microenvironment: focus on mesenchymal stem cells and myeloid derived suppressor cells. Histol Histopathol 26: 941–951. 21630223 10.14670/HH-26.941

[14] Goldstein RH, Reagan MR, Anderson K, Kaplan DL, Rosenblatt M (2010) Human bone marrow-derived MSCs can home to orthotopic breast cancer tumors and promote bone metastasis. Cancer Res 70: 10044–10050. 21159629 10.1158/0008-5472.CAN-10-1254 PMC3017423

[15] Chaturvedi P, Gilkes DM, Wong CC, Kshitiz, Luo W, et al (2013) Hypoxia-inducible factor-dependent breast cancer-mesenchymal stem cell bidirectional signaling promotes metastasis. J Clin Invest 123: 189–205. 23318994 10.1172/JCI64993 PMC3533298

[16] Torsvik A, Bjerkvig R (2013) Mesenchymal stem cell signaling in cancer progression. Cancer Treat Rev 39: 180–188. 22494966 10.1016/j.ctrv.2012.03.005

[17] Gottschling S, Granzow M, Kuner R, Jauch A, Herpel E, et al (2013) Mesenchymal stem cells in non-small cell lung cancer–different from others? Insights from comparative molecular and functional analyses. Lung Cancer 80: 19–29. 23294501 10.1016/j.lungcan.2012.12.015

[18] Karnoub AE, Dash AB, Vo AP, Sullivan A, Brooks MW, et al (2007) Mesenchymal stem cells within tumour stroma promote breast cancer metastasis. Nature 449: 557–563. 17914389 10.1038/nature06188

[19] Koh BI, Kang Y (2012) The pro-metastatic role of bone marrow-derived cells: a focus on MSCs and regulatory T cells. EMBO Rep 13: 412–422. 22473297 10.1038/embor.2012.41 PMC3343352

[20] Eramo A, Lotti F, Sette G, Pilozzi E, Biffoni M, et al (2008) Identification and expansion of the tumorigenic lung cancer stem cell population. Cell Death Differ 15: 504–514. 18049477 10.1038/sj.cdd.4402283

[21] Bertolini G, Roz L, Perego P, Tortoreto M, Fontanella E, et al (2009) Highly tumorigenic lung cancer CD133+ cells display stem-like features and are spared by cisplatin treatment. Proc Natl Acad Sci U S A 106: 16281–16286. 19805294 10.1073/pnas.0905653106 PMC2741477

[22] Wang S, Xu ZY, Wang LF, Su W (2013) CD133+ cancer stem cells in lung cancer. Front Biosci 18: 447–453. 10.2741/4113 23276935

[23] Singh SK, Hawkins C, Clarke ID, Squire JA, Bayani J, et al (2004) Identification of human brain tumour initiating cells. Nature 432: 396–401. 15549107 10.1038/nature03128

[24] Gu P, Harwood LJ, Zhang X, Wylie M, Curry WJ, et al (2007) Isolation of retinal progenitor and stem cells from the porcine eye. Mol Vis 13: 1045–1057. 17653049 PMC2776542

[25] Micalizzi DS, Farabaugh SM, Ford HL (2010) Epithelial-mesenchymal transition in cancer: parallels between normal development and tumor progression. J Mammary Gland Biol Neoplasia 15: 117–134. 20490631 10.1007/s10911-010-9178-9 PMC2886089

[26] Guarino M, Rubino B, Ballabio G (2007) The role of epithelial-mesenchymal transition in cancer pathology. Pathology 39: 305–318. 17558857 10.1080/00313020701329914

[27] Zhao JJ, Pan K, Wang W, Chen JG, Wu YH, et al (2012) The prognostic value of tumor-infiltrating neutrophils in gastric adenocarcinoma after resection. PLoS One 7: e33655. 22442706 10.1371/journal.pone.0033655 PMC3307751

[28] Talmadge JE (2011) Immune cell infiltration of primary and metastatic lesions: mechanisms and clinical impact. Semin Cancer Biol 21: 131–138. 21145968 10.1016/j.semcancer.2010.12.002

[29] Zhang BC, Gao J, Wang J, Rao ZG, Wang BC, et al (2011) Tumor-associated macrophages infiltration is associated with peritumoral lymphangiogenesis and poor prognosis in lung adenocarcinoma. Med Oncol 28: 1447–1452. 20676804 10.1007/s12032-010-9638-5

[30] Mantovani A, Allavena P, Sica A, Balkwill F (2008) Cancer-related inflammation. Nature 454: 436–444. 18650914 10.1038/nature07205

[31] Lazennec G, Richmond A (2010) Chemokines and chemokine receptors: new insights into cancer-related inflammation. Trends Mol Med 16: 133–144. 20163989 10.1016/j.molmed.2010.01.003 PMC2840699

[32] Germano G, Allavena P, Mantovani A (2008) Cytokines as a key component of cancer-related inflammation. Cytokine 43: 374–379. 18701317 10.1016/j.cyto.2008.07.014

[33] Davies PS, Powell AE, Swain JR, Wong MH (2009) Inflammation and proliferation act together to mediate intestinal cell fusion. PLoS One 4: e6530. 19657387 10.1371/journal.pone.0006530 PMC2716548

[34] Johansson CB, Youssef S, Koleckar K, Holbrook C, Doyonnas R, et al (2008) Extensive fusion of haematopoietic cells with Purkinje neurons in response to chronic inflammation. Nat Cell Biol 10: 575–583. 18425116 10.1038/ncb1720 PMC4230437

[35] Nygren JM, Liuba K, Breitbach M, Stott S, Thorén L, et al (2008) Myeloid and lymphoid contribution to non-haematopoietic lineages through irradiation-induced heterotypic cell fusion. Nat Cell Biol 10: 584–592. 18425115 10.1038/ncb1721

[36] Cogle CR, Theise ND, Fu D, Ucar D, Lee S, et al (2007) Bone marrow contributes to epithelial cancers in mice and humans as developmental mimicry. Stem Cells 25(8): 1881–1887. 17478582 10.1634/stemcells.2007-0163

