# Supplementary figures and images for: EMT and Acquisition of Stem Cell-Like Properties Are Involved in Spontaneous Formation of Tumorigenic Hybrids between Lung Cancer and Bone Marrow-Derived Mesenchymal Stem Cells
Source: PLoS One. 2014 Feb 6;9(2):e87893. doi: 10.1371/journal.pone.0087893 (PMC3916343; doi:10.1371/journal.pone.0087893)

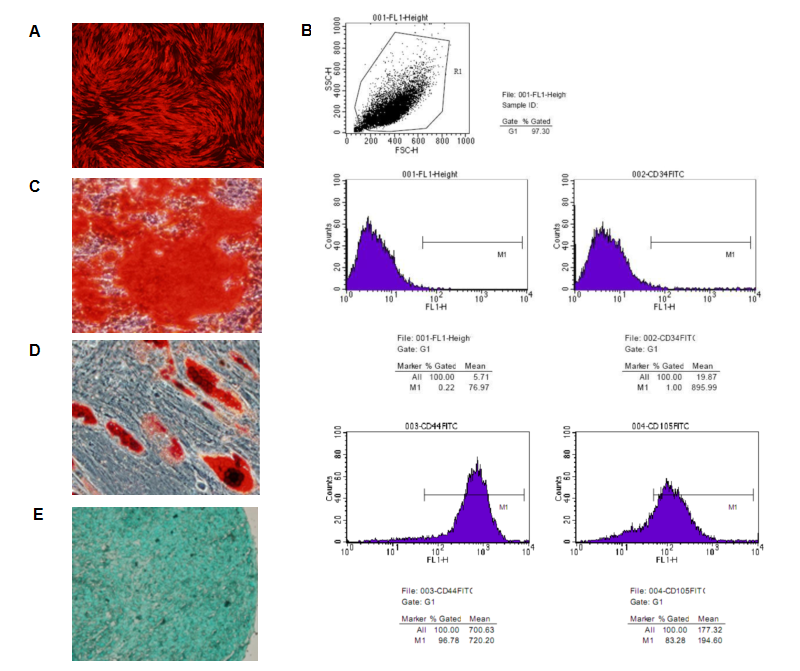

Supplement: Figure S1 — Characterization of MSCs. (A) Cultured MSCs exhibited fibroblast-like cell morphology. (B) Expression of CD44, CD105 and CD34 on MSC-lung cancer hybrids using flow cytometry. (C) Osteoblasts differention. (D) Adipocytes differention. (E) Chondrocytes differentiation. Scale bar, 25 µm. (TIF) [file pone.0087893.s001.tif]

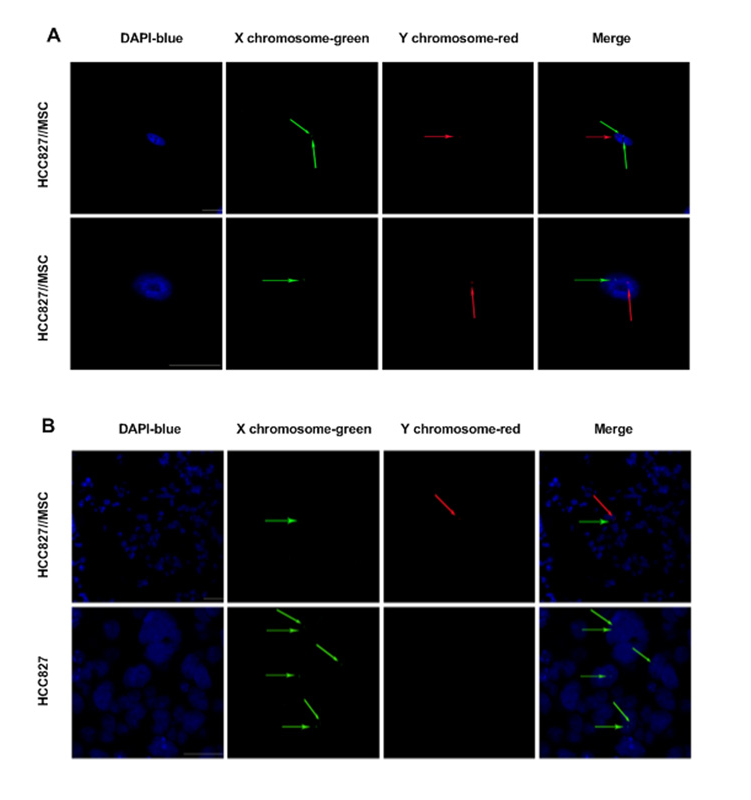

Supplement: Figure S2 — FISH analysis of HCC827/MSC hybrids. (A) Male MSCs were cultured with female HCC827 lung cancer cells for 8 days and fixed. FISH (Spectrum red-Y chromosome and Spectrum green-X chromosome) was performed and nuclei were stained with DAPI (blue). Green arrows show X chromosomes and red arrows, Y chromosomes. Upper pictures show an HCC827/MSC hybrid harboring one nucleus with one Y chromosome and two X chromosomes; Lower pictures show an HCC827/MSC hybrid harboring one nucleus with one Y chromosome and one X chromosome. (B) Spontaneously-formed male-derived tumorigenic hybrids were detected in vivo by FISH. Tumor specimens were derived from tumors generated by subcutaneous injection of mixed MSCs and HCC827 cells (above) or from parental tumor (below) in NOD/SCID mice. Scale bar, 25 µm. (TIF) [file pone.0087893.s002.tif]

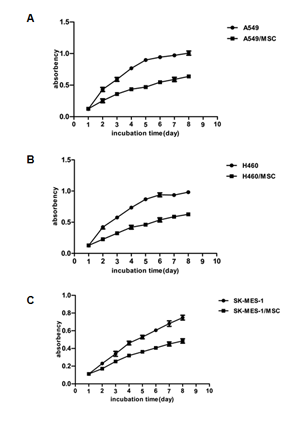

Supplement: Figure S3 — Growth curves of hybrids and respective parental lung cancer cells. A CCK-8 assay was performed and the absorbance were detected at 450 nm. (A) Growth curves of A549/MSC hybrids and A549. (B) Growth curves of H460/MSC hybrids and H460. (C) Growth curves of SK-MES-1/MSC hybrids and SK-MES-1. The data are reported as mean ± SEM of three independent experiments performed in triplicate. Asterisks depict statistically-significant differences between the heterotypic hybrids and respective parental lung cancer cells (*P<0.01). (TIF) [file pone.0087893.s003.tif]

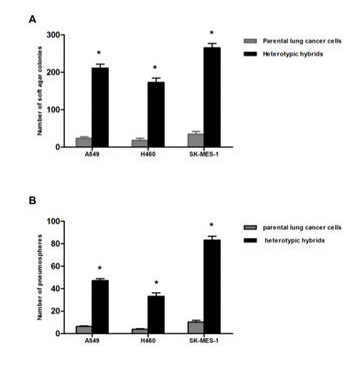

Supplement: Figure S4 — Tumor sphere formation ability of heterotypic hybrids and respective parental lung cancer cells. (A) Soft agar assays: Single cells (1×10^5^ per well) were plated into soft agar in 6-well plates in triplicate. Quantification of heterotypic hybrids and respective parental A549, H460 or SK-MES-1 cells. (B) Secondary mammosphere quantification of heterotypic hybrids and respective parental A549, H460 or SK-MES-1 cells. Primary spheres were dispersed by trypsinization and replated at 1×10^3^ cells/well. The pneumospheres were cultured for 5–7 days, then pneumospheres with diameter larger than 50 µm were counted. n = 12. Data are reported as mean ± SEM. Asterisks indicate statistically-significant differences between the heterotypic hybrids and their respective parental non-small-cell lung cancer cells (*P<0.001). (TIF) [file pone.0087893.s004.tif]
