# Supplementary material for: EMT and Acquisition of Stem Cell-Like Properties Are Involved in Spontaneous Formation of Tumorigenic Hybrids between Lung Cancer and Bone Marrow-Derived Mesenchymal Stem Cells
Source: PLoS One. 2014 Feb 6;9(2):e87893. doi: 10.1371/journal.pone.0087893 (PMC3916343; doi:10.1371/journal.pone.0087893)
Supplement: Table S1 — Forward and reverse primer sequences using real-time PCR. (DOC) [file pone.0087893.s005.doc]

**Table S1. Forward and reverse primer sequences using real-time PCR.**

| **Gene** | **Forward primer** | **Reverse primer** |
| --- | --- | --- |
| *Twist* | GTCCGCAGTCTTACGAGGAG | TGGAGGACCTGGTAGAGGAA |
| *Snail1* | GGTTCTTCTGCGCTACTGCT | TAGGGCTGCTGGAAGGTAAA |
| *Slug* | GGGGAGAAGCCTTTTTCTTG | TCCTCATGTTTGTGCAGGAG |
| *ZEB1* | GCACAAGAAGAGCCACAAGTA | GCAAGACAAGTTCAAGGGTTC |
| *ZEB2* | TTCCTGGGCTACGACCATAC | TGTGCTCCATCAAGCAATTC |
| *hFOXC2* | GCCTAAGGACCTGGTGAAGC | TTGACGAAGCACTCGTTGAG |
| *Notch1* | CCGCCTTTGTGCTTCTGTTC | CCGCCTTTGTGCTTCTGTTC |
| *ALDH1* | GGCAGCCATTTCTTCTCA | TGTCCAAGTCGGCATCAG |
| *Bmi1* | CTCCACCTCTTCTTGTTTG | CTGATGACCCATTTACTGA |
| *CD133* | TCTTGACCGACTGAGACCCAAC | ACTTGATGGATGCACCAAGCAC |
| *Oct4* | GTATTCAGCCAAACGACCAT | GCTTCCTCCACCCACTTCT |
| *SOX2* | CGATGCCGACAAGAAAACTT | CAAACTTCCTGCAAAGCTCC |
| *Nanog* | CAGAAGGCCTCAGCACCTAC | ATTGTTCCAGGTCTGGTTGC |
| *Vimentin* | CCAGGCAAAGCAGGAGTC | GGGTATCAACCAGAGGGAGT |
| *E-cadherin* | TGCCCAGAAAATGAAAAAGG | GTGTATGTGGCAATGCGTTC |
| *Fibronectin* | AGCCTCGAAGAGCAAGAGG | CAAAACTTCAGCCCCAACTT |
| *α-SMA* | GAGAAGAGTTACGAGTTGCCTGA | TGTTAGCATAGAGGTCCTTCCTG |
| *GAPDH* | GACCTGACCTGCCGTCTA | GGAGTGGGTGTCGCTGT |
